# Autologous non‐human primate model for safety assessment of *piggyBac* transposon‐mediated chimeric antigen receptor T cells on granulocyte–macrophage colony‐stimulating factor receptor

**DOI:** 10.1002/cti2.1207

**Published:** 2020-11-22

**Authors:** Hirokazu Morokawa, Shigeki Yagyu, Aiko Hasegawa, Miyuki Tanaka, Shoji Saito, Hidemi Mochizuki, Kengo Sakamoto, Akihito Shimoi, Yozo Nakazawa

**Affiliations:** ^1^ Department of Pediatrics Shinshu University School of Medicine Matsumoto Japan; ^2^ Center for Advanced Research of Gene and Cell Therapy Shinshu University School of Medicine Matsumoto Japan; ^3^ Department of Pediatrics, Graduate School of Medical Science Kyoto Prefectural University of Medicine Kyoto Japan; ^4^ Ina Research Inc. Ina Japan; ^5^ Institute for Biomedical Sciences Interdisciplinary Cluster for Cutting Edge Research Shinshu University Matsumoto Japan

**Keywords:** CAR T cells, hGMR, non‐human primate, off‐tumor toxicity, cynomolgus macaque

## Abstract

**Objectives:**

Chimeric antigen receptor (CAR)‐T cell therapy redirected to specific antigens on tumor cells is a promising immunotherapy strategy for various cancers. Most target antigens are also expressed on normal tissues at varying levels, and therefore, a considerable challenge in the field is determining safety profiles, including life‐threatening off‐tumor and off‐target toxicities. The granulocyte–macrophage colony‐stimulating factor receptor (hGMR) is a promising target for CAR T‐cell therapy for a subset of acute myelocytic leukaemia, although it is also expressed on normal cells including monocytes, macrophages, CD34‐positive haematopoietic cells and vascular endothelial cells. hGMR and other immune‐related proteins are highly conserved between humans and cynomolgus macaques (*Macaca fascicularis*). Therefore, in this study, we engineered cynomolgus T cells to express CAR molecules redirected to hGMR by *piggyBac* (PB) transposon‐based gene transfer and adoptively transferred autologous hGMR‐CAR T cells into cynomolgus macaques.

**Methods:**

We established PB‐mediated human GMR (hGMR)‐specific CAR T cells using cynomolgus peripheral blood mononuclear cells and transferred them into autologous individuals, and evaluated the potential toxicity related to hGMR‐CAR T cells.

**Results:**

hGMR‐CAR T cells did not exert overt organ toxicities such as bone marrow suppression, monocytopenia and vasculitis, although they recognised and killed cynomolgus monocytes and macrophages *in vitro*.

**Conclusion:**

Although our model did not simulate a tumor‐bearing model, it supports the safety of hGMR‐CAR T cells and demonstrates the usefulness of a non‐human primate model to evaluate the safety of T‐cell products by assessing off‐tumor/off‐target toxicity before clinical trials.

## Graphical abstract

We pre‐clinically evaluated the safety of granulocyte–macrophage colony‐stimulating factor (GMCSF) receptor (GMR)–chimeric antigen receptor (CAR) T cells. For this purpose, we used a novel immunocompetent non‐human primate (NHP) model, established using bioengineered cynomolgus T cells expressing CAR‐T molecules redirected against the GMR by *piggyBac* transposon‐based (PB) gene transfer. Our study provides evidence of the potential safety of GMR‐CAR T cells and the utility of an NHP model for elucidating the efficacy and safety of T‐cell products before their introduction into clinical trials.

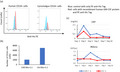

## Introduction

Chimeric antigen receptor (CAR) T‐cell therapy redirected to specific antigens on tumor cells is a promising treatment strategy for relapsed/refractory tumors, which cannot be cured by current standard treatments.^1^, ^2^ CAR T‐cell therapy specific to the CD19 molecule has achieved considerable success in a subset of patients with highly refractory B‐cell tumors,^3^, ^4^, ^5^, ^6^ and various CAR T‐cell products are being extended to treat other cancers including myeloid malignancies^7^ and solid tumors.^8^

Despite the clinical success of CAR T‐cell therapy for leukaemia, early clinical trials of CD19 CAR T‐cell therapy have elucidated considerable and often life‐threatening toxicities.^9^, ^10^, ^11^ Some major toxicities are cytokine release syndrome (CRS) and immune effector cell‐associated neurotoxicity syndrome (ICANS), which are characterised by profound immune cell reactions, irrespective of whether they are caused by CAR‐T or bystander recipient immune cells^12^; they occur following the secretion of inflammatory cytokines. Another serious toxicity owing to the ‘on‐target/off‐tumor’ or ‘off‐target’ effect is an unintended attack on normal tissues by CAR T cells.^13^ Ideally, the target antigens of genetically modified T cells should be exclusively expressed on tumor cells; however, most targets are antigens that are commonly expressed on normal cells. Furthermore, even when these common antigens are expressed at extremely low levels on normal cells, severe toxicities could occur when these antigens are recognised by T cells. A clinical trial of CAR T cells targeting human epidermal growth factor receptor 2 (HER2) reported one such case, where a patient experienced acute respiratory distress within 15 min and died 5 days after T‐cell infusion.^14^ The pathogenesis of this condition involved a massive alveolar injury and haemorrhagic microangiopathy caused by the recognition of HER2 expressed at a low level by CAR T cells on lung epithelial cells.^14^ This observation suggests that tumor‐specific antigens or neo‐antigens could be ideal candidates to reduce these toxicities. However, there is a risk of unexpected promiscuous recognition of unrelated antigens/epitopes derived from a normal protein. Linette *et al*.^13^ described a lethal 'off‐target' cardiovascular toxicity in patients with malignant melanoma treated with melanoma‐associated antigen 3 (MAGE‐A3)‐specific T‐cell receptor (TCR) T cells. This toxicity was induced by the unpredicted promiscuous cross‐reaction of MAGE‐A3‐specific TCR with titin‐derived human leucocyte antigen‐A1‐presented peptide expressed on cardiomyocytes.^15^ Therefore, off‐tumor/off‐target cross‐reactivity must be thoroughly studied before clinical trials.

Tumor xenograft‐immunodeficient murine models have been widely used to evaluate the antitumor effects of immune effector cells. However, these models are not suitable to evaluate immune cell‐related toxicities because of the low cross‐reactivity of human immune cells with murine proteins. Non‐human primate (NHP) models could provide a more appropriate platform because most NHP proteins are highly conserved. The usefulness of NHP models has been demonstrated in studies on the safety of immune‐effector cells, including virally engineered CAR T cells.^16^, ^17^

Previously, we developed *piggyBac* transposon (PB)‐mediated CAR T cells redirected to the human granulocyte–macrophage colony‐stimulating factor (GM‐CSF) receptor (hGMR),^18^ which is highly expressed in subtypes of myeloid malignancies, and revealed their antitumor efficacy in a murine xenograft model.^19^ The hGMR is expressed on normal cells, including monocytes, macrophages, CD34‐positive haematopoietic cells^18^ and vascular endothelial cells, at varying levels. Therefore, hGMR‐specific CAR could exert unwanted killing effects on hGMR‐expressing cells or even off‐target toxicity via the cross‐reaction of hGMR‐CAR T cells with hGMR derivatives on normal cells.

This was a pre‐clinical study on the safety of PB‐hGMR‐CAR T cells using an immunocompetent NHP model. Because the amino acid sequence of the hGMR and immune‐related proteins, including effector cytokines, is highly conserved between cynomolgus macaques and humans (Supplementary figure 1), we genetically engineered cynomolgus T cells to express hGMR‐specific CAR and evaluated the potential toxicity related to hGMR‐CAR T cells.

## Results

### Production and characterisation of cynomolgus hGMR‐CAR T cells for adoptive transfer

**Figure 1:**
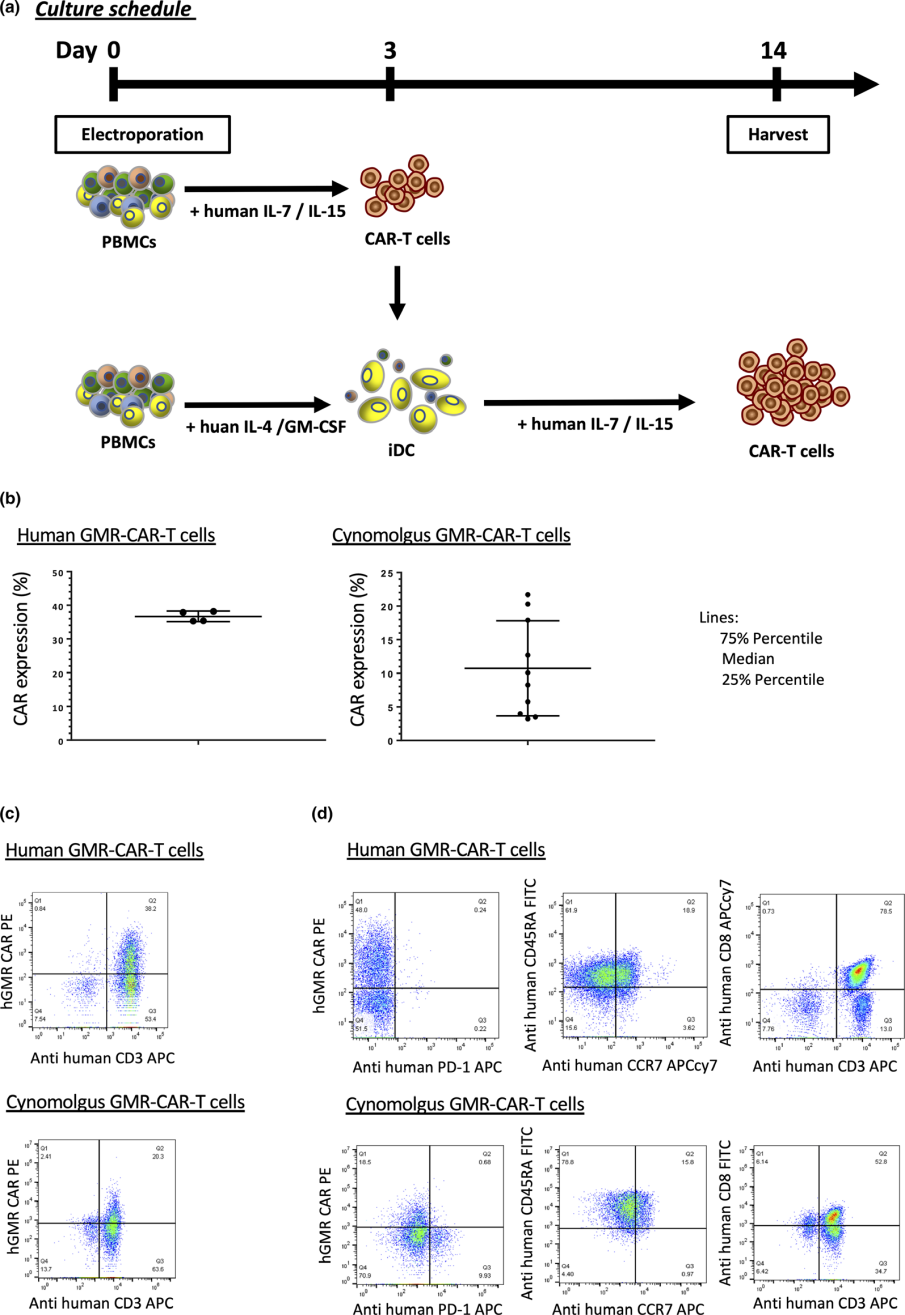
**(a)** Schematic representation of the generation method of PB‐modified hGMR‐CAR T cells. PBMCs were transfected with hGMR‐CAR via the *piggyBac* transposon system. On day 3, the cells were cocultured with iDCs derived from PBMCs with interleukin (IL)‐4 and GM‐CSF for 72 h. The electroporated T cells were cultured with IL‐7 and IL‐15. Fourteen days after culture initiation, cells were harvested and analysed. **(b)** Expression of human or cynomolgus hGMR‐CAR T cells. **(c)** Representative flow cytometry results each cynomolgus and human hGMR‐CAR T cells. **(d)** Characterisation of cynomolgus hGMR‐CAR T cells.

hGMR‐CAR T cells generated from human and cynomolgus macaques using the PB transposon system are shown in Figure 1a. We optimised a previously established production procedure for PB‐mediated‐CAR T cells from human peripheral blood mononuclear cells (PBMCs).^20^ We consistently obtained approximately 20% cynomolgus CD3^+^/CAR^+^ cynomolgus T cells (3.21–21.7%, median 9.17%, *n* = 10; Figure 1b and c), and this was slightly lower than that of human PBMC‐derived hGMR‐CAR T cells produced using the same protocol (35.3–38.2%, median 36.65%, *n* = 4; Figure  1b and c). Immunophenotyping showed that cynomolgus hGMR‐CAR T cells were CD8‐dominant and that the stem cell memory T‐cell phenotype was enriched, as characterised by CD45RA^+^/CCR7^+^ and lower PD‐1 expression compared with the control cells. This observed phenotype was consistent with that of human hGMR‐CAR T cells (Figure 1d). These results indicated that the characteristics of PB‐modified cynomolgus hGMR‐CAR T cells were similar to those of human hGMR‐CAR T cells.

### Cynomolgus hGMR‐CAR T cells recognised and killed hGMR‐positive human cells and cynomolgus monocytes and M1 macrophages *in vitro*

**Figure 2:**
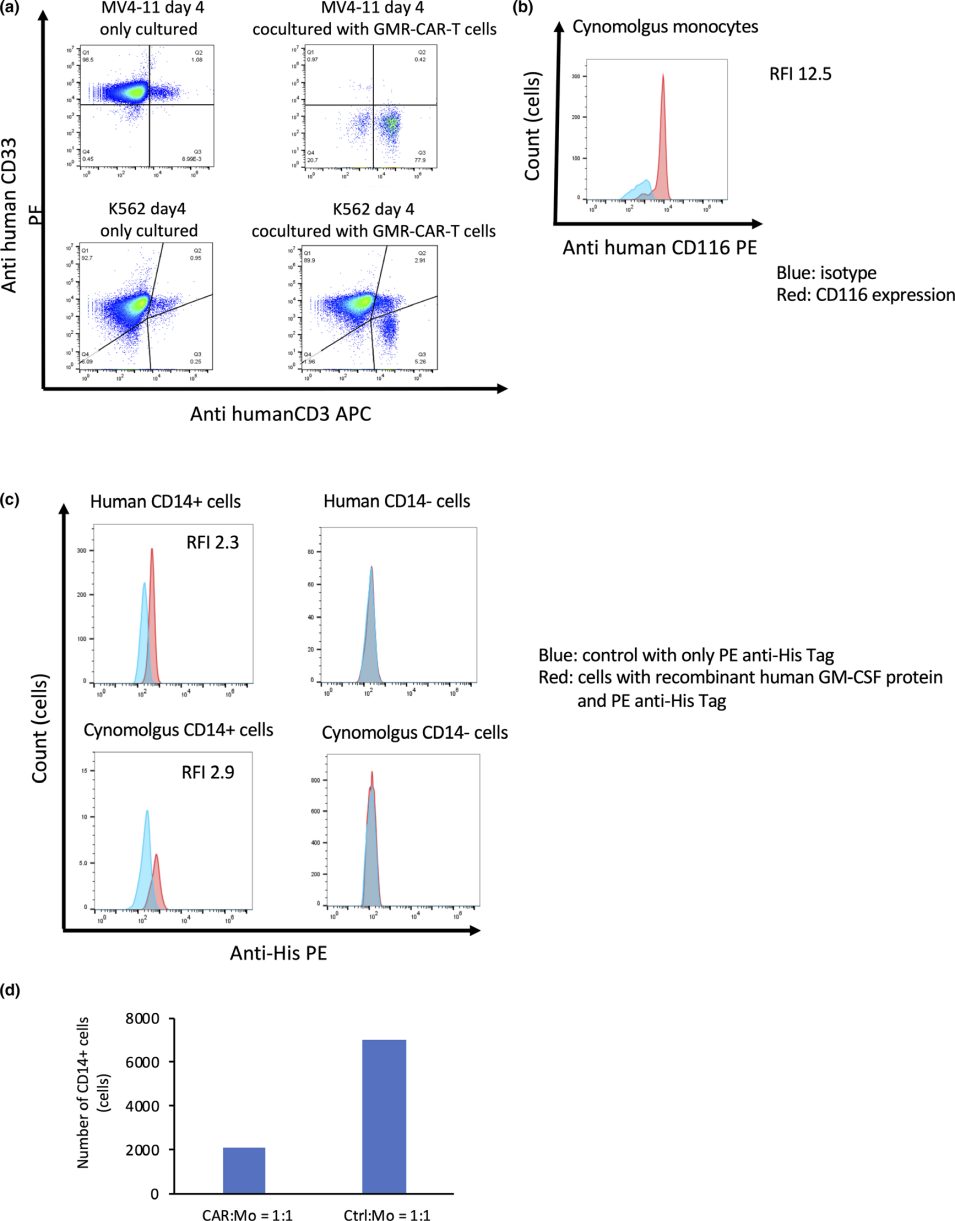
Cynomolgus hGMR‐CAR T cells exhibited potent antitumor activity against hGMR + tumor and cynomolgus hGMR + normal cells. **(a)** Cynomolgus hGMR‐CAR T cells were cocultured with MV4‐11 or K562 at effector: target (E:T) ratios of 1:1 for 4 days and analysed by flow cytometry. **(b)** Cynomolgus GMRs expressed on cynomolgus monocytes. Blue lines exhibit isotype, and red lines exhibit CD116 expression on monocytes or M1 macrophages. **(c)** Recombinant human GM‐CSF proteins combined with cynomolgus and human GMR on CD14‐positive cells. Red histogram indicates the cells bound to recombinant human GM‐CSF protein. Blue histogram indicates a control sample stained with only PE anti‐His‐tag. **(d)** Cynomolgus hGMR‐CAR‐T or control T (Ctrl) cells were cocultured with cynomolgus monocytes (Mo) at an E:T ratio of 1:1.

Cynomolgus hGMR‐CAR T cells showed substantial killing potency against MV4‐11 cells, but no toxic effect was observed in K562 cells on day 4 after coculture (Figure 2a). Furthermore, we confirmed that the human CD116 antibody can bind to cynomolgus GMRs (cGMRs) expressed on cynomolgus monocytes (Figure 2b), indicating a highly conserved structure between hGMR and cGMR. In contrast, cGMR was not strongly expressed in cynomolgus M1 macrophages as determined by flow cytometry (Supplementary figure 2). To explore whether human GM‐CSF can recognise cGMR, cynomolgus CD14‐positive monocytes were stained with recombinant human GM‐CSF proteins. Human GM‐CSF proteins could recognise cynomolgus CD14‐positive monocytes at levels similar to human CD14‐positive monocytes (median fluorescence intensity of 2.9 and 2.3, respectively) (Figure 2c). This indicates that hGMR‐CAR T cells, which have the hGM‐CSF extracellular domain as an antigen recognition site, recognised cynomolgus and human CD14‐positive cells. As anticipated, cynomolgus hGMR‐CAR T cells killed cynomolgus CD14‐positive monocytes (Figure 2d). Cynomolgus hGMR‐CAR T cells also reduced the number of cynomolgus M1 macrophages despite their low expression of cGMR as determined by flow cytometry (Supplementary figure 2). Overall, these data indicate the killing potency of cynomolgus hGMR‐CAR T cells and potential off‐tumor effects of hGMR‐CAR T cells on cGMR‐expressing normal cells, including monocytes and M1 macrophages.

### Adoptive transfer of cynomolgus hGMR‐CAR T cells exerted no overt toxicities

**Figure 3:**
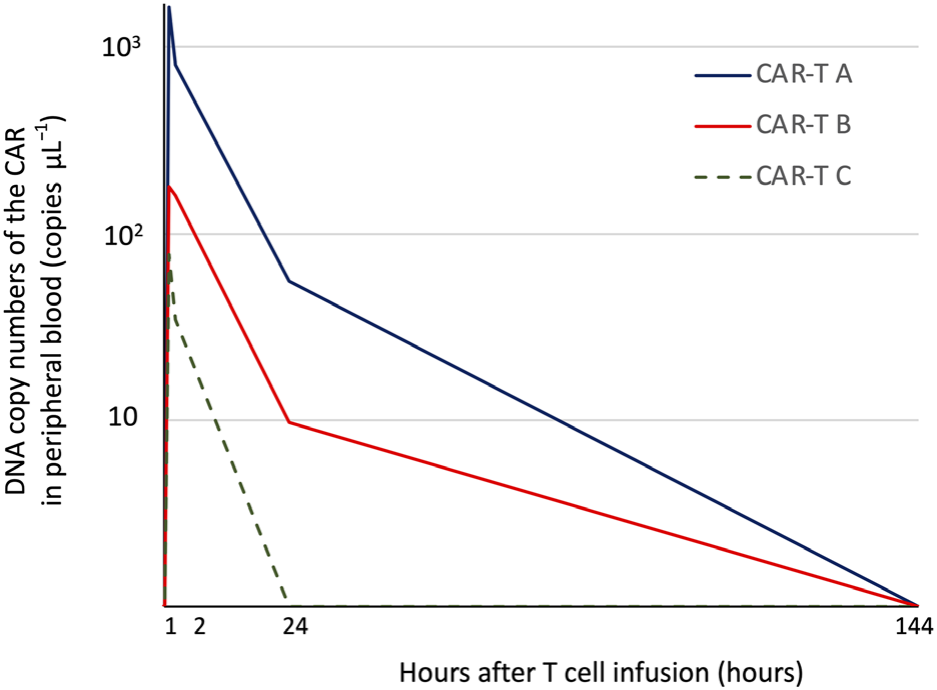
Kinetics of cynomolgus hGMR‐CAR T cells. The qPCR analysis of hGMR‐CAR transgene sequences confirmed the persistence of hGMR‐CAR T cells in all three macaques at 1 h after infusion, which gradually declined to undetectable levels by day 6.

**Figure 4:**
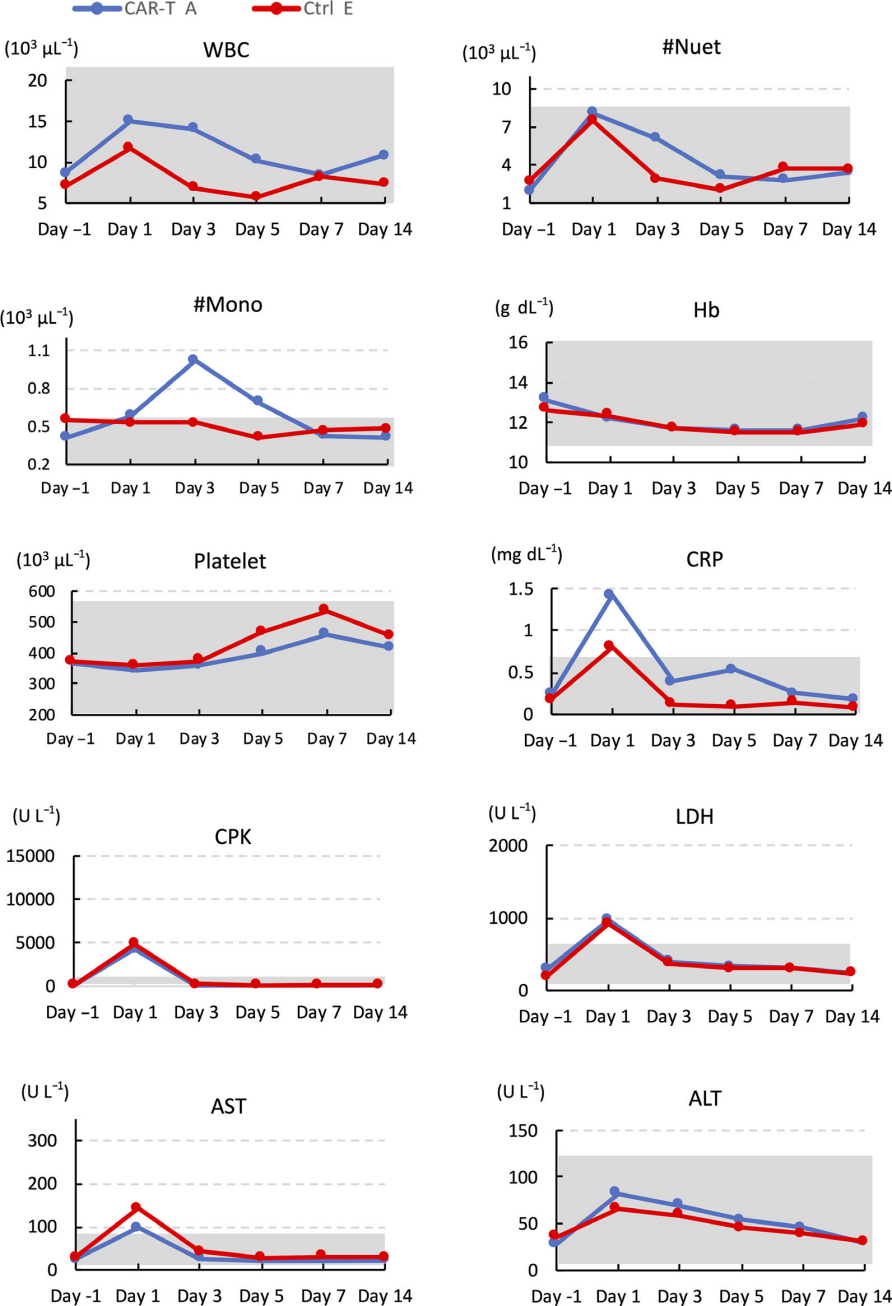
Representative data of complete blood counts and blood chemistry of macaque treated with CAR‐T (A) and control T cell (Ctrl) (E) before and at the indicated days after adoptive transfer. The grey‐shaded area demarks the macaque‐specific normal range for each parameter, which is shown in Supplementary figure 2 in detail. ALT, alanine transaminase; AST, aspartate transaminase; CPK, creatine phosphokinase; CRP, C‐reactive protein; Hb, haemoglobin; LDH, lactate dehydrogenase; Mono, monocyte; Neut, neutrophil; WBC, white blood cell.

The qPCR analysis of hGMR‐CAR transgene sequences confirmed the persistence of hGMR‐CAR T cells in all three macaques at 1 h after infusion, and it gradually declined to undetectable levels by day 7 (Figure 3); this limited the analysis of long‐term toxicity. In addition, immediate or delayed clinical abnormalities were not observed at this cell dose, including the general conditions, body weight and food consumption (data not shown). Respiratory symptoms are potential clinical manifestations because alveolar macrophages could express cGMR, but there were no signs of respiratory distress during the investigational period. The blood cell count remained within the normal range according to the in‐house reference data (Supplementary figure 3) throughout the study period (Figure 4, Supplementary figure 4). However, overt myelosuppression owing to the impaired function of cGMR‐expressing cynomolgus haematopoietic cells recognised by hGMR‐CAR T cells might be a concern. Of note, the cynomolgus monocyte count did not decrease after CAR T‐cell infusion, although cynomolgus hGMR‐CAR T cells killed cynomolgus monocytes in our *in vitro* study (Figure 2c).

A transient elevation in creatine phosphokinase, lactate dehydrogenase, aspartate transaminase and C‐reactive protein levels was observed in both groups; however, this was not an hGMR‐CAR‐specific change. It can be attributed to muscular damages induced during blood sampling from macaques in the restrainer (Figure 4, Supplementary figure 4). Other serum biochemistry analyses revealed no considerable changes in both groups (data not shown). The coagulation test also remained within the normal limit with no elevation in the D‐dimer (Supplementary figure 5), although vascular endothelial cells also expressed cGMR. This suggested that the trivial expression of cGMRs on vascular endothelial cells did not induce off‐tumor toxicity of hGMR‐CAR T cells in this model. Overall, overt organ toxicity related to hGMR‐CAR T cells was not observed during the investigational period.

### Cynomolgus hGMR‐CAR T cells were transiently and mildly activated *in vivo* but did not induce overt CRS

**Figure 5:**
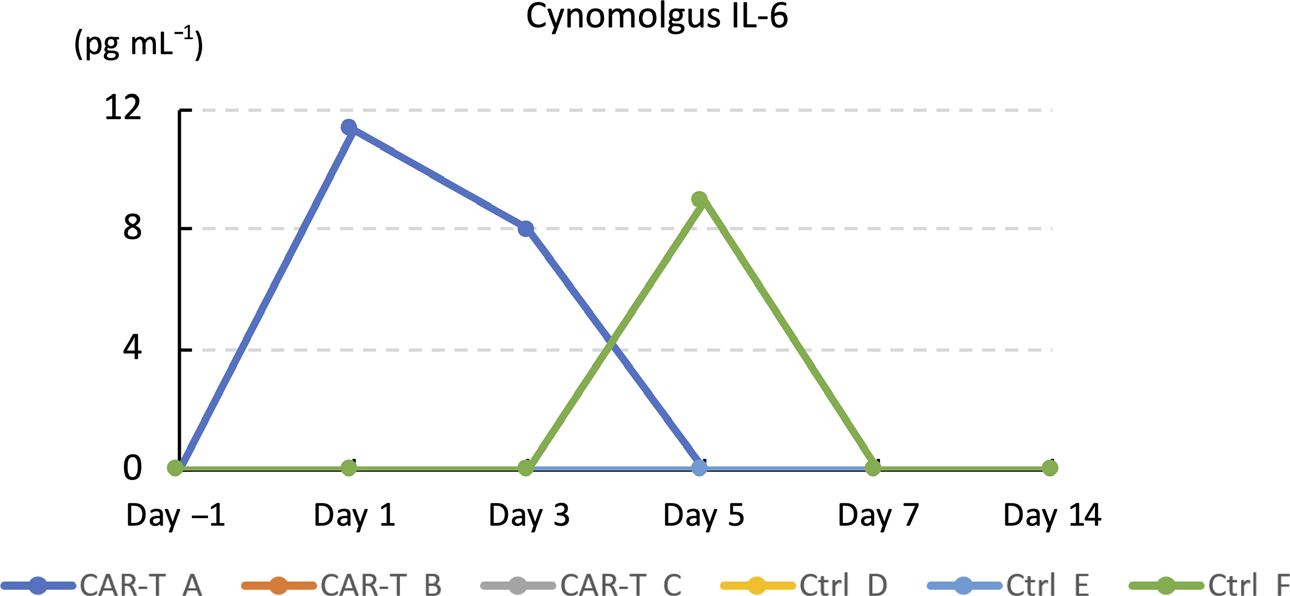
Plasma interleukin (IL)‐6 levels before and at the indicated days after adoptive transfer of cynomolgus hGMR‐CAR‐T or control T cells.

To determine whether hGMR‐CAR T cells induce cytokine release, we sequentially evaluated inflammatory cytokine profiles. The results revealed no pathological elevation in the cynomolgus TNF‐α, IFN‐γ, IL‐1β, IL‐2 and GM‐CSF levels during the investigational period (data not shown). However, a transient and mild increase in cynomolgus IL‐6 level was observed on day 2 in a CAR T‐cell‐infused macaque that showed the highest copy number of the *CAR* transgene in the peripheral blood, indicating the transient activation of CAR T cells *in vivo*. However, this macaque did not develop any symptoms related to CRS.^13^ We did not observe a robust expansion of CAR T cells *in vivo*, which is often evident in patients with a high tumor burden and related to CRS incidence.^16^ One macaque in the control group showed a modest and transient elevation in human IL‐6 on day 6 (Figure 5), and this was considered non‐specific because most of the adoptive T cells disappeared according to the kinetics study (Figure 3).

### No pathological changes were observed in the recipients of cynomolgus hGMR‐CAR T cells

The assessment of potential pathological changes in macaque tissues showed no macroscopic changes in the heart, brain, lung, kidney, liver, spleen, spinal cord, skin and bone marrow. Moreover, there were no microscopic changes related to the CAR T‐cell infusion (Supplementary table 1), although the infiltration of cynomolgus mononuclear cells was detected in the liver and kidney, which is normally observed in cynomolgus macaques.^21^, ^22^

## Discussion

In this study, we successfully generated PB‐mediated cynomolgus PBMC‐derived CAR T cells redirected against the hGMR. The cynomolgus T cells expressing hGMR‐CAR removed hGMR overexpressed on human acute myeloid leukaemia (AML) cells, cynomolgus monocytes *in vitro*. This indicated that cynomolgus hGMR‐CAR T cells were functional in both human and cynomolgus GMR‐expressing cells. The human and cynomolgus immunoreceptor tyrosine‐based activation motifs of CD3ζ are identical, and other key cytotoxic cytokines, including IFN‐γ, perforin and granzyme B, are highly conserved with more than 95% identity (data not shown). This observation suggests that the killing potency of cynomolgus hGMR‐CAR T cells was directed against both human and cynomolgus hGMR‐expressing cells. There were no considerable clinical toxicities, including monocytopenia and coagulopathies. Our findings support the usefulness of the NHP model to evaluate the safety of CAR T cells redirected to tumor antigens, which are highly conserved between humans and macaques. To the best of our knowledge, this is the first pre‐clinical safety study of PB‐mediated CAR T cells using an NHP model.

Tumor antigens for immunotherapy should be ideally expressed only in tumor tissue, and little or none is desirable in normal tissues. However, most antigens currently targeted by CAR T cells or monoclonal antibodies are expressed in normal and tumor tissues at various levels. Tumor antigens co‐expressed in non‐vital tissues could be alternative targets for immunotherapies. For example, although CD19 is recognised by CAR T cells^3^, ^4^, ^5^, ^6^ or a bispecific monoclonal antibody,^23^ it demonstrated clinical success in the immunotherapy of refractory B‐cell malignancies, because it is exclusively expressed in the B‐cell lineage. Nevertheless, normal B‐cell aplasia and the subsequent agammaglobulinaemia are the most frequent off‐tumor effects of CD19‐targeted immunotherapy, and they can be treated by repeated administration of intravenous gamma globulin without a life‐threatening infection.^24^ Because of the absence of specific antigen for myeloid malignancies, CAR T cells for AML have also been developed to target non‐specific antigens, including Lewis Y,^25^ CD33,^26^ CD123,^27^ fms‐related receptor tyrosine kinase 3^28^ and C‐type lectin‐like molecule,^29^, ^30^, ^31^ which are also expressed in the subset of haematopoietic stem/progenitor cells. For example, targeting CD33 in AML results in toxicities, which destroy normal myeloid cells,^26^ and these toxicities are attributed to the co‐expression of CD33 in haematopoietic stem cells at low levels. GMRs are also expressed on monocytes, dendritic cells, vascular endothelial cells and haematopoietic stem/progenitor cells, and consequently, monocytopenia, coagulopathy and disruption of bone marrow function would be potential off‐tumor effects of the systemic administration of hGMR‐CAR T cells. Indeed, cynomolgus monocytes were recognised and eradicated by hGMR‐CAR T cells in our *in vitro* study, and this suggests sufficient cGMR expression on cynomolgus monocytes to activate hGMR‐CAR T cells. However, signs of monocytopenia were not observed following the systemic administration of hGMR‐CAR T cells, indicating that hGMR‐CAR T cells did not damage monocytes in the blood despite the expression of cGMR on the cell surface. This discrepancy could be explained by differences in the affinity of cGMR on activated and quiescent monocytes with hGM‐CSF.^18^ Our hGMR‐CAR utilised the natural hGMR ligand (GM‐CSF) as an antigen‐binding site,^18^ and GM‐CSF preferentially interacted with the active form of GMR, which consisted of the GM‐CSFR alpha and beta complexes.^32^ Thus, hGMR‐CAR T cells modestly interacted with quiescent monocytes or other cGMR‐expressing normal cells *in vivo*. Another possible reason for the lack of *in vivo* toxicity is the difference in the E:T ratio between *in vitro* and *in vivo* studies. In fact, a recent study demonstrated that ROR1‐specific CAR T cells at a high dose induce a higher response to the antigen by elevating cynomolgus IFN‐γ and IL‐6 levels *in vivo*, whereas at a low dose, it showed no considerable elevation in inflammatory cytokines.^16^ Moreover, a lack of persistence of infused T cells could be a limitation of this therapy if immune cell‐associated adverse effects, such as CRS, occur in response to a massive expansion/persistence of effector T cells.^33^ Because our study design did not establish a tumor‐bearing (antigen‐presenting cell bearing) NHP model,^16^ the infused CAR T cells may not be fully activated as frequently observed in patients who experience CRS, which is related to a high tumor burden and CAR T‐cell overactivation.^34^ Nevertheless, on‐target/off‐tumor or off‐target toxicities usually develop within a few hours after T‐cell infusion,^14^ and our NHP study could be useful for evaluating the possible occurrence of unexpected damage to normal organs caused by the cross‐reaction of CAR T cells on weakly expressed cGMR or its derivatives.

An autologous NHP model may enable the evaluation of the kinetics and long‐term effects of cell products without the concern of immune rejection and reproduction of the host immunological reaction to cellular products. Nevertheless, some potential limitations of the NHP model, including the differences in the distribution of the antigen on normal NHP tissues, should be carefully considered. Consequently, this model cannot precisely represent the effects of CAR T cells if the expression profile of the target antigen differs considerably between humans and NHPs. Furthermore, another possible limitation in the evaluation of CAR‐T‐specific toxicity is there is no tumor‐bearing NHP model, and the antitumor potency and safety could not be simultaneously evaluated in the same individuals, as the incidence of CRS or ICANS is associated with the tumor burden.^35^ Another limitation of this study was the lack of optimisation study to determine the infused cell dose. Although the infused cell dose utilised in clinical trials targeting myeloid malignancy ranged from approximately 1 × 10^5^/kg to 1 × 10^6^/kg, we chose the infused cell dose as 3 × 10^5^/kg in this study, as a relatively low infused cell dose might lead to the underestimation of the potential off‐tumor toxicities. Therefore, a dose–escalation study is warranted to estimate the possible toxicities induced by GMR‐CAR T‐cell infusion at high doses. Finally, although cynomolgus macaques and humans have a high homology, the results obtained from cynomolgus macaques may not be entirely extrapolatable to humans. Importantly, the quality of CAR T cells derived from human or cynomolgus PBMCs might differ in the transduction efficiency of the CAR transgene or phenotype of the final product. In fact, the CAR expression in cynomolgus hGMR‐CAR T cells was lower than that in human hGMR‐CAR T cells, although they were produced using the same electroporation protocol.

In conclusion, we evaluated the safety of hGMR‐CAR T cells using an NHP model and demonstrated that hGMR‐CAR T cells had minimal effects on normal tissue. The NHP system is a promising tool to evaluate the possible occurrence of unexpected off‐tumor/off‐target toxicity of cells used in genetically modified immune cell‐based therapy, and non‐clinical safety studies using this NHP model may validate the clinical applicability of PB‐hGMR‐CAR T cells.

## Methods

### Plasmid

The PB transposase plasmid (pCMV‐PB) used in this study has been described previously.^18^ The pIRII‐hGMR‐CAR plasmid encoding the hGMR‐binding domain derived from GM‐CSF fused to the human CD28 intracellular portion and human CD3ζ chain is described elsewhere in detail.^18^

### Generation of hGMR‐CAR T cells using cynomolgus PBMCs

Animal experiments were performed with protocols approved by the Shinshu University School of Medicine and Ina Research Inc., Institutional Animal Care and Use Committee (Approval Number: 19021). Cynomolgus macaques were housed in Ina Research Inc. (Ina, Japan), which is accredited by the Association for Assessment and Accreditation of Laboratory Animal Care International. Four female and two male cynomolgus macaques aged 4–5 years from Vietnam or China were used in this study. We collected PBMCs from 20 mL of blood samples from macaques and generated PB‐modified hGMR‐CAR T cells.

Approximately 20 × 10^6^ of cynomolgus PBMCs were freshly isolated from peripheral blood using Ficoll‐Paque Plus (GE Healthcare, Chicago, IL) diluted to 90% with PBS (FUJIFILM, Tokyo, Japan), and the contaminant red blood cells (RBCs) were lysed using eBioscience™ 1 × RBC lysis buffer (Thermo Fisher Scientific Inc., Waltham, MA). Cynomolgus PBMCs were electroporated with 7.5 μg each of pIRII‐hGMR‐CAR and pCMV‐PB using NEPA21 Super Electroporator (Nepagene, Inc., Chiba, Japan). The electroporated T cells were cultured in complete culture medium (CCM) consisting of ALyS™ 705 supplemented with 5% artificial serum (both from Cell Science and Technology Institute Inc., Miyagi, Japan), and recombinant human interleukin (IL)‐7 and IL‐15 (at a final concentration of 10 and 5 ng mL^–1^, respectively; Miltenyi Biotec, Bergisch Gladbach, Germany) in multiwell 48‐well plates at 37°C in a humidified 5% CO_2_ incubator. On day 3, the cells were cocultured with immature dendritic cells (iDCs) derived from autologous cynomolgus PBMCs with human IL‐4 and human GM‐CSF (at a final concentration of 10 ng mL^−1^ each, Miltenyi Biotec) in ALyS™ 705 for 72 h. These cells were maintained in CCM, transferred to the Gas Permeable Rapid Expansion (G‐REX^®^; Wilson Wolf, Saint Paul, MN) platform on day 6 and then harvested for further experiments on day 14 after culture initiation (Figure 1a).

### Generation of cynomolgus‐ activated T cells

Cynomolgus T cells without genetic modifications were generated and used as the control T cells. Cynomolgus PBMCs were electroporated with only 7.5 μg of pCMV‐PB and cultured in CCM. On day 2, these cells were transferred to a plate coated with purified mouse anti‐human CD3 (BD Pharmingen, Franklin Lakes, NJ) and CD28 (Miltenyi Biotec) antibodies, incubated in CCM for 48 h and then expanded using the G‐REX platform.

### Cell line

hGMR alpha (hCD116)‐positive human MV4‐11 leukaemia and hCD116‐negative K562 cell lines were purchased from American Type Culture Collection (ATCC, Manassas, VA) and were maintained in Roswell Park Memorial Institute (RPMI)‐1640 medium (Thermo Fisher Scientific Inc.), supplemented with 10% foetal bovine serum (FBS; Cytiva, Marlborough, MA) at 37°C in a humidified 5% CO_2_ incubator.

### Phenotypic analysis of cynomolgus hGMR‐CAR T cells

Cell proliferation was determined by counting the cells using automated cell counter R1 (Olympus, Tokyo, Japan). The expression of CAR on the cell surface was determined using phycoerythrin (PE) anti‐human GM‐CSF and PE anti‐human CD33 (both from Miltenyi Biotec); allophycocyanin (APC) anti‐human CD3, peridinin–chlorophyll–protein (PerCP) anti‐human CD4, PerCP anti‐human CD8, fluorescein isothiocyanate anti‐human CD45RA, PE anti‐human CD56 and PE anti‐human CD62L (all from BD Pharmingen); PE anti‐human CD116 (Beckman Coulter, Pasadena, CA); and APC anti‐human CD197 [C‐C chemokine receptor type 7 (CCR7)] and APC anti‐human programmed cell death 1 (PD‐1; both from BioLegend, San Diego, CA) to characterise the phenotype of CAR T cells and examine the cell lines for coculture experiments. All flow cytometry data were acquired using BD fluorescent‐activated cell‐sorting Accuri C6 Plus and analysed using FlowJo v10.5 software (both from BD Biosciences, Franklin Lakes, NJ).

### Phenotypic analysis of human hGMR‐CAR T cells

The expression of human hGMR‐CAR T cells on the cell surface was determined using APC anti‐human CD3 (Miltenyi Biotec), APCcy7 anti‐human CCR7 and APCcy7 anti‐human CD8 (BioLegend). All other reagents were the same as those used in the cynomolgus phenotypic analysis.

### Interaction of human GM‐CSF with human and cynomolgus GMR

Human and cynomolgus PBMCs were preincubated with Human BD Fc Block™ (BD Pharmingen), per the manufacturer’s protocol, and then, the cells were stained with recombinant human GM‐CSF protein (His‐tag) (Sino Biological Inc., Peking, China), PE anti‐His‐tag (BioLegend) and APC anti‐human CD14 (BioLegend). The expression of cell surface proteins was analysed using FlowJo software. Relative fluorescence intensity (RFI) was measured as a ratio of the geometric mean fluorescence intensity of specific markers to the geometric mean fluorescence intensity of isotype controls.

### Cytotoxicity assay

Tumor (MV4‐11 or K562) cells were cocultured at a density of 2 × 10^5^ with cynomolgus hGMR‐CAR‐T or control T cells at an effector:target (E:T) ratio of 1:1 in RPMI‐1640 medium supplemented with 10% FBS in a 48‐well plate. Before coculture and on day 4, the cells were harvested and analysed by flow cytometry to quantify live leukaemia and T cells using Count Bright Absolute Counting Beads^®^ (Invitrogen, Carlsbad, CA). Monocytes were separated from cynomolgus PBMCs using human CD14 MicroBeads (Miltenyi Biotec). M1 macrophages were differentiated from CD14^+^ monocytes using the Human M1 Macrophage Differentiation Kit (R & D systems, Minneapolis, MN). Monocytes and M1 macrophages were also cocultured with hGMR‐CAR T cells, and live monocytes were acquired on days 0 and 2 as described above.

### Animal protocols and monitoring

**Figure 6:**
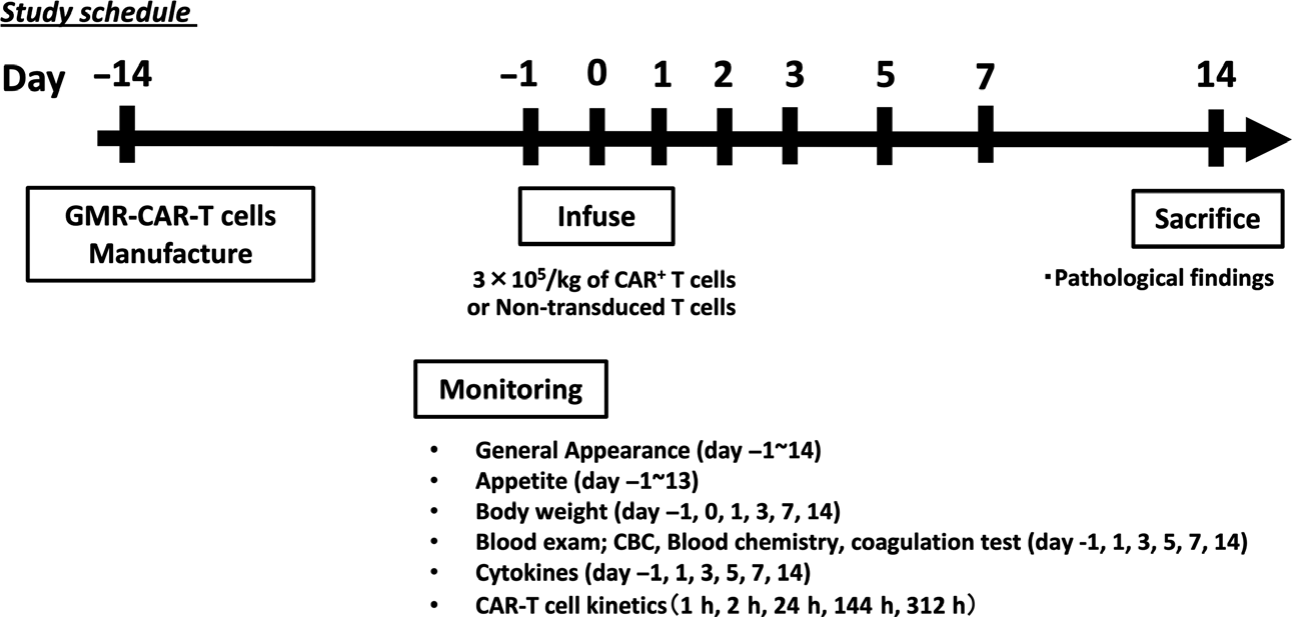
Study schedule of the toxicity test of autologous hGMR‐CAR T cells using macaques.

We generated cynomolgus hGMR‐CAR‐T and control‐activated T cells from three macaques each, which were intravenously administered either matched autologous hGMR‐CAR‐T or control T cells. The infused cells were adjusted to a density of 3 × 10^5^ CAR T cells kg^–1^ according to the positivity of hGMR‐CAR (total number of infused T cells; 1.47, 7.56 and 9.30 × 10^6^ cells kg^−1^). The number of infused control T cells was comparable with the number of total T cells administered to the CAR‐T‐treated group. All macaques were closely monitored for clinical manifestations, such as the changes in general conditions, food consumption and body weight. Additionally, we performed sequential sampling of blood for complete cell counts and coagulation tests using XN‐2000 and CA‐150, respectively (Sysmex Inc., Hyogo, Japan), blood chemistry using 7180 Clinical Analyzer (Hitachi High‐Tech Technologies, Tokyo, Japan), cytokine profiling and kinetic analysis to determine CAR T‐cell persistence (Figure 6). All macaques were anaesthetised by intravenous injection of thiopental sodium and were painlessly euthanised by exsanguination from the axillary and femoral arteries and veins on day 14 after T‐cell infusion for pathological evaluation.

### Cytokine analysis of plasma samples

Blood samples were obtained on days −1, 1, 3, 5, 7 and 14 after CAR T‐cell infusion. Plasma samples were extracted and stored at −80°C before cytokine analysis and subsequently examined for cynomolgus IL‐1β, IL‐2, IL‐6, IL‐8, interferon (IFN)‐γ, tumor necrosis factor (TNF)‐α and GM‐CSF using Bio‐Plex 200 (Bio‐Rad Laboratories Inc., Hercules, CA) and an NHP cytokine magnetic bead panel (EMD Millipore, Burlington, MA).

### Kinetic analysis of CAR T‐cell persistence

Blood samples were collected at 1, 2, 24, 144 and 312 h after CAR T‐cell infusion into ethylenediaminetetraacetic acid disodium‐containing tubes and stored at −80°C before analysis. Genomic DNA was extracted using the DNeasy Blood and Tissue Kit (Qiagen Inc., Hilden, Germany). Polymerase chain reaction (PCR) of the specific sequence of pIRII‐hGMR‐CAR was conducted using the ViiA™ 7 real‐time PCR system (Thermo Fisher Scientific Inc.), TaqMan Universal Master Mix II with UNG (Thermo Fisher Scientific Inc.) and the following primer and probe sets: forward, 5′‐ACGTGACTTTTAAGATTTAACTCATACGA‐3′; reverse, 5′‐CAATGAATAATATGGCTAATGGCCA‐3′; and probe, 5′‐FAM‐CTTGTTATAGATAAGATCTTC‐MGB‐3′.

### Pathological analysis

On day 14 after CAR T‐cell infusion, the heart, spleen, liver, gallbladder, kidneys, brain, and thoracic spinal cord were harvested from each euthanised macaque, fixed with 10% formaldehyde and stained with haematoxylin and eosin. All pathological samples were carefully examined by trained pathologists.

### Statistical analysis

Chimeric antigen receptor expression data are presented as median with 25% and 75% percentiles and as mean ± SD, calculated using GraphPad PRISM 7 (GraphPad Software, San Diego, CA). Because of the small number of macaques used in the safety test, we did not conduct statistical analyses.

## Conflict of interest

Hidemi Mochizuki, Kengo Sakamoto and Akihito Shimoi are employees of Ina Research, Inc. The authors have no other conflicts of interest.

## Author contributions

**Hirokazu Morokawa:** Data curation; Formal analysis; Investigation; Writing‐original draft; Writing‐review & editing. **Shigeki Yagyu:** Conceptualization; Formal analysis; Investigation; Methodology; Project administration; Supervision; Writing‐original draft; Writing‐review & editing. **Aiko Hasegawa:** Data curation; Formal analysis; Investigation; Methodology; Writing‐review & editing. **Miyuki Tanaka:** Data curation; Formal analysis; Investigation; Methodology; Writing‐review & editing. **Shoji Saito:** Resources; Writing‐review & editing. **Hidemi Mochizuki:** Conceptualization; Data curation; Formal analysis; Investigation; Methodology; Writing‐review & editing. **Kengo Sakamoto:** Data curation; Formal analysis; Investigation; Methodology; Writing‐review & editing. **Akihito Shimoi:** Conceptualization; Data curation; Formal analysis; Investigation; Methodology; Project administration; Resources; Writing‐review & editing. **Yozo Nakazawa:** Conceptualization; Formal analysis; Funding acquisition; Investigation; Methodology; Project administration; Resources; Supervision; Writing‐review & editing.
