# Supplementary material for: Autologous non‐human primate model for safety assessment of piggyBac transposon‐mediated chimeric antigen receptor T cells on granulocyte–macrophage colony‐stimulating factor receptor
Source: Clin Transl Immunology. 2020 Nov 22;9(11):e1207. doi: 10.1002/cti2.1207 (PMC7680920; doi:10.1002/cti2.1207)
Supplement: Supplementary file 1 [file CTI2-9-e1207-s001.pdf]

Supporting Information

Supplementary figure 1

Amino-acid sequences of the GMR in cynomolgus macaque and human.

Supplementary figure 2

a. Cynomolgus hGMRs expressed on M1 macrophages. Blue lines exhibit isotype and red lines exhibit CD116 expression on M1 macrophages. b. Cynomolgus hGMR-CAR-T or control T (Ctrl) cells were cocultured with cynomolgus M1 macrophages (M1) at an E:T ratio of 1:1.

Supplementary figure 3

In-house data of blood examination. Two to four-years-old healthy cynomolgus macaques housed in Ina Research Inc. from 2016 to 2019 were subjected to a blood examination to obtain the reference values. The minimum and maximum values of each parameter were determined.

n; number of macaques analyzed, HGB; hemoglobin, WBC; white blood cell, Mono; monocyte, Neut; neutrophil, Mono; monocyte, AST; aspartate transaminase, ALT; alanine transaminase, LDH; lactate dehydrogenase, CPK; creatine phosphokinase, CRP; C-reactive protein

Supplementary figure 4

Complete blood counts and blood chemistry of all macaques before and at the indicated days after adoptive transfer cynomolgus GMR-CAR-T cells.

WBC; white blood cell, Neut; neutrophil, Lym lymphocyte, Mono; monocyte, Hb;

hemoglobin, Retic; reticulocyte, CRP; C-reactive protein, CPK; creatine phosphokinase, LDH; lactate dehydrogenase, AST; aspartate transaminase, ALT; alanine transaminase

Supplementary figure 5

Coagulation tests of all macaques before and at the indicated days after adoptive transfer cynomolgus GMR-CAR-T cells.

PT; prothrombin time, APTT; activated partial thromboplastin time

Supplementary table 1.

There were no microscopic changes in the liver, gall bladder, kidney, spleen, heart, brain, and thoracic spinal cord due to CAR-T cell infusion, although infiltration of cynomolgus mononuclear cells was detected in the liver and kidney, and this is a normal change.

Corresponding Author; Shigeki Yagyu

Supplementary figure 1

```
MACACA MWLQGLLLLGTVACSISAPARSPSPGTQPWEHVNAIQEARRLLNLSRDTAAEMNKTVEVV  60
_HUMAN MWLQSLLLLGTVACSISAPARSPSPSTQPWEHVNAIQEARRLLNLSRDTAAEMNETVEVI
MACACA SEMFDLQEPSCLQTRLELYKQGLQGSLTKLKGPLTMMASHYKQHCPPTPETSCATQIITF  120
_HUMAN SEMFDLQEPTCLQTRLELYKQGLRGSLTKLKGPLTMMASHYKQHCPPTPETSCATQIITF
MACACA QSFKENLKDFLLVIPFDCWEPVQE
_HUMAN ESFKENLKDFLLVIPFDCWEPVQE
```

Supplementary figure 2

Corresponding Author; Shigeki Yagyu

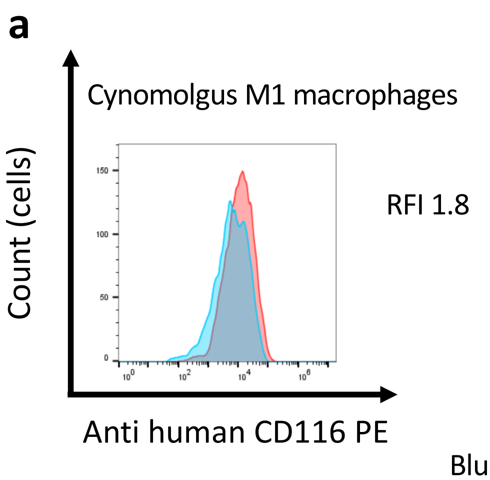

Blue: isotype
Red: CD116 expression

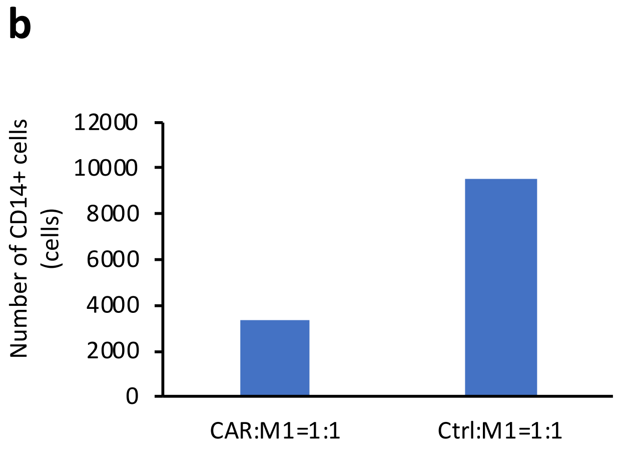

Supplementary figure 3

Corresponding Author; Shigeki Yagyu

Parameters	(Unit)	n	Min	Max
HGB	(g/dL)	525	10.8	15.9
WBC	(10^3/μL)	524	3.48	22.08
Neut	(10^3/μL)	518	0.47	11.27
Mono	(10^3/μL)	522	0.14	1.11
PLT	(10^3/μL)	525	157	595

Parameters	(Unit)	n	Min	Max
AST	(U/L)	901	13	81
ALT	(U/L)	902	8	122
LDH	(U/L)	780	141	581
CPK	(U/L)	838	58	1316
CRP	(mg/dL)	93	0.01	0.64

Supplementary figure 4

Corresponding Author; Shigeki Yagyu

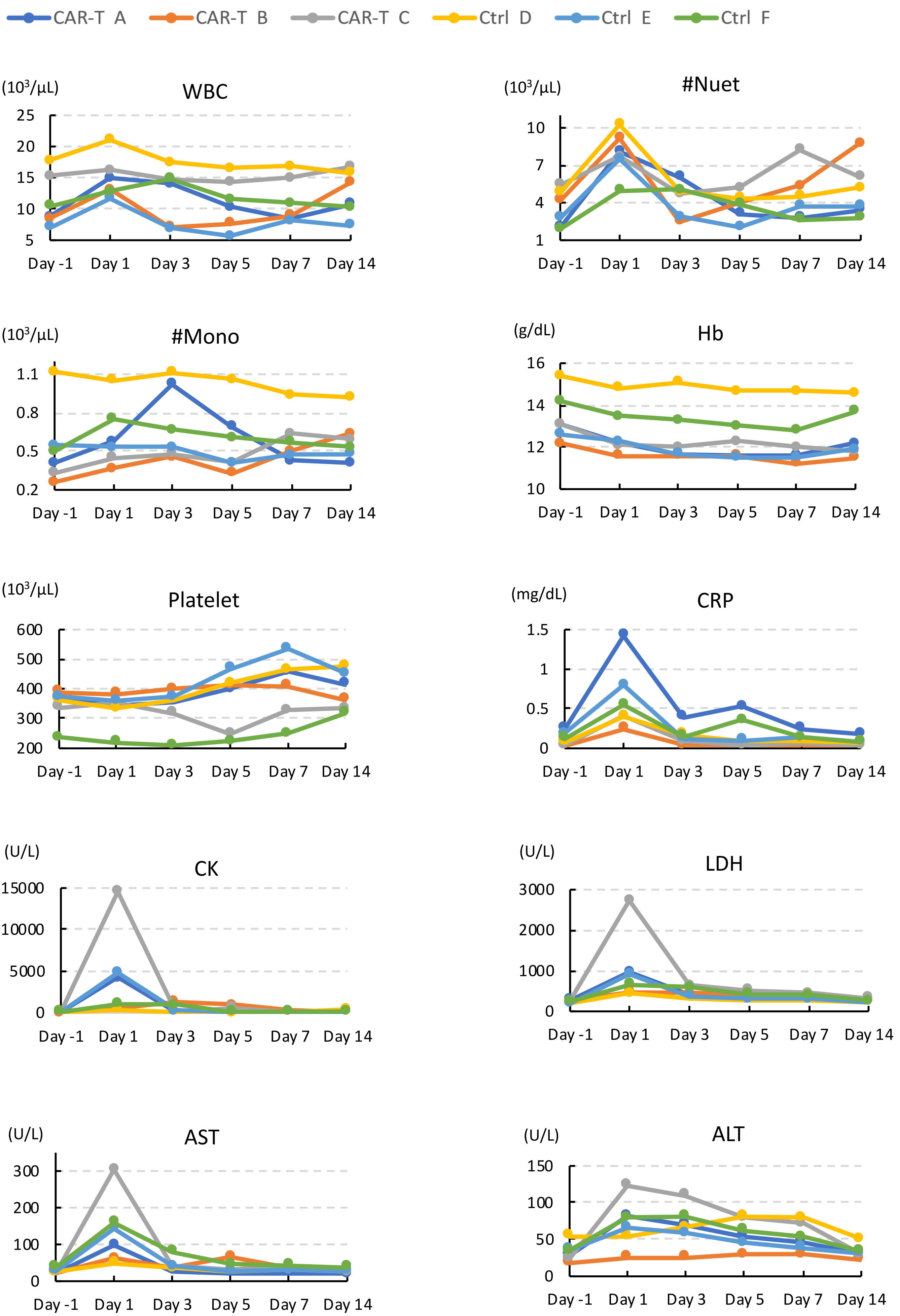

Supplementary figure 5

Corresponding Author; Shigeki Yagyu

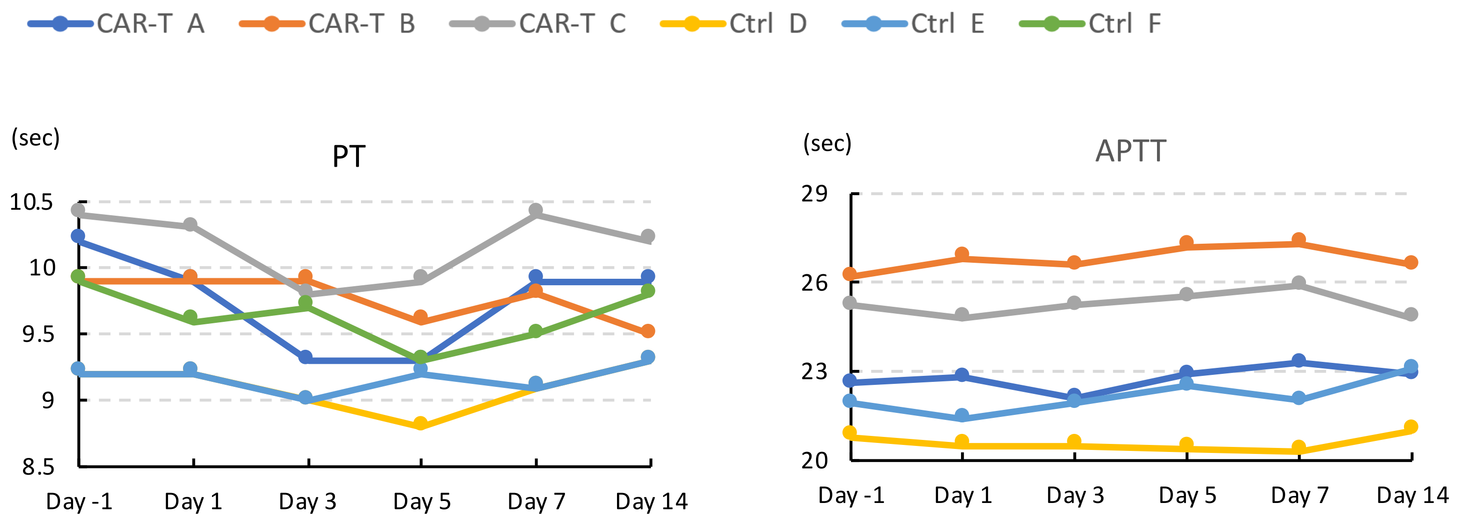

D-dimer (ng/mL)

	CAR-T A	CAR-T B	CAR-T C	Ctrl D	Ctrl E	Ctrl F
Day -1	<200	<200	200-500	<200	<200	<200
Day 1	<200	200-500	200-500	200-500	500<	200-500
Day 3	200-500	<200	200-500	500<	200-500	200-500
Day 5	500<	200-500	200-500	500<	200-500	<200
Day 7	200-500	200-500	200-500	500<	200-500	200-500
Day 14	<200	200-500	200-500	500<	500<	<200

Supplementary table 1

Corresponding Author; Shigeki Yagyu

Organ	Findings	Macaque		
		CAR-T A	CAR-T B	CAR-T C
Liver	*infiltrate, mononuclear cell*	minimal	minimal	-
Gallbladder	Pathological change	-	-	-
Kidney	*infiltrate, mononuclear cell*	minimal	mild	minimal
Spleen	Pathological change	-	-	-
Heart	Pathological change	-	-	-
Brain	Pathological change	-	-	-
Spinal cord (thoracic)	Pathological change	-	-	-
